# Neuroprotective Effect of Radix Trichosanthis Saponins on Subarachnoid Hemorrhage

**DOI:** 10.1155/2015/313657

**Published:** 2015-05-18

**Authors:** Ying Chen, Haiyan Sun, Liyong Huang, Juxiang Li, Wenke Zhou, Jingling Chang

**Affiliations:** ^1^College of Life Science, Henan Normal University, Xinxiang, Henan 453007, China; ^2^School of Life Science and Technology, Henan Institute of Science and Technology, Xinxiang, Henan 453003, China; ^3^Department of Neurosurgery, the First Affiliated Hospital of Xinxiang Medical University, Weihui, Henan 453100, China

## Abstract

Redox homeostasis has been implicated in subarachnoid hemorrhage (SAH). As a result, antioxidants and/or free radical scavengers have become an important therapeutic modality. Considering that radix trichosanthis (RT) saponins exhibited strong antioxidant ability both *in vivo* and *in vitro*, the present study aimed to reveal whether the neuroprotective activities of RT saponins were mediated by p38/p53 signal pathway after SAH. An established SAH model was used and superoxide dismutase (SOD), malondialdehyde (MDA), induced nitric oxide synthase (iNOS), nitric oxide (NO), lactate dehydrogenase (LDH), p-p38, and p53 activation were detected after 48 h of SAH. The results showed that RT saponins inhibited iNOS expression to restore NO to basal level. Moreover, compared with Cu/Zn-SOD, RT saponins (2 mg/kg/d dosage) significantly increased Mn-SOD activity after SAH. Accompanied with lowered NO and elevated SOD, decreased p38 phosphorylation and p53 activities were observed, especially for RT saponins at 2 mg/kg/d dosage. In this setting, the neurological outcome was also improved with less neuronal cells damage after RT saponins pretreatment. Our findings demonstrated the beneficial effects of RT saponins in enhancing neuroprotective effects by deducing iNOS activity, normalizing SOD level, and inhibiting p-p38 and p53 expression, hence offering significant therapeutic implications for SAH.

## 1. Introduction

Stroke is a leading cause of morbidity and mortality worldwide. Even though subarachnoid hemorrhage (SAH) accounts for only 5–10% of all strokes, it is a major devastating subtype affecting 30,000 people in North America annually [1]. There is a growing body of evidence that the imbalance of the reactive oxygen species (ROS) and reactive nitrogen species (RNS) and the antioxidant defense system may be associated with neurological deficits and poor outcome of SAH [2–5]. Superoxide dismutase (SOD), including mitochondrial located Mn-SOD and cytoplasm dispersed Cu/Zn-SOD isoforms, is a well-studied antioxidant enzyme to eliminate intracellular ROS. RNS, composed of nitric oxide (NO) and its ramification ONOO^−^, was produced mainly by induced nitric oxide synthase (iNOS) under pathological conditions. Under this context, downregulated endogenous antioxidant systems and overexpressed iNOS were observed both in experimental and in clinical SAH studies [6, 7].

p38, one of mitogen-activated protein kinases (MAPK) family, plays pivotal roles in apoptosis, cell cycle progression, cell growth, and differentiation. In neurodegenerative diseases, p38 has been found to reduce ROS and RNS through overexpressed SOD and decreased iNOS, whereas others showed that oxidative stress can also activate proapoptotic p38/p53 axis [8–11]. Moreover, upregulation of p53 has, in turn, been identified to suppress antioxidant genes and proapoptotic genes, such as* MnSOD* and* BAX* [12, 13]. As a result, oxidative stress associated p38/p53 pathway has been found to associate with blood-brain barrier (BBB) permeability, brain edema, inflammation, and cell death [14, 15]. More importantly, several groups have reported that inhibition p38 overexpression was proved to be more beneficial in improving neurological recovery in experimental models after SAH, partly due to scavenging abundant ROS and RNS, and inhibiting apoptosis [11, 16].

Indeed, it is well documented that pharmacological agents will have promising prospect if they could inhibit multiple injurious mechanisms. Saponins, the most abundant secondary components of glycosidic nature, have attracted attention in recent years due to their broad-spectrum functions. It has long been known that saponins have neuroprotective effects via antioxidant and antiapoptosis pathways [17–19]. Radix trichosanthis (RT), the dry root tuber of* Trichosanthis kirilowii* Maxim (Cucurbitaceae), is an extensive used traditional Chinese medicine functioning as antitumor, antivirus, immunomodulatory, and abortifacient medicine. Up to now, trichosanthin and fructus trichosanthis have been widely studied; however, little is known about the biological effects of RT saponins. Based on our previous report that RT saponins have strong antioxidant activities both* in vitro* and* in vivo*, we deduced that RT saponins might have a neuroprotective effect [20]. In the present study, we try to improve our understanding of whether RT saponins have neuroprotective effects on* in vivo* SAH models or not and the involved mechanisms, mainly focusing on oxidative stress associated proapoptotic p38/p53 pathway.

## 2. Experimental Procedures

### 2.1. Plant Materials

RT used in this study was collected from farmland of Henan Institute Science and Technology in autumn of 2012. RT was authenticated by Professor Li Meng at the Department of Botany, Henan Institute Science and Technology.

### 2.2. Preparation and Extraction of the Crude Saponins

Samples were dried at room temperature and milled into dry powder. Ethanol, n-butanol, and ethyl acetate (EtOAc) were used for the extraction [20]. Briefly, 200 g of RT powder was extracted three times with 10-fold ethanol. After ethanol solvent was removed by a rotary evaporator, the dried ethanol extract was then dissolved in hot water and partitioned successively with petroleum ether. After defatted, equal volumes of n-butanol and EtOAc were used and collected the extraction. Using a column-layer chromatographic silica gel (160–200 mesh) (C116888, Aladdin, China), only the fraction eluted with 80% ethanol was collected. The total saponins content was determined by the vanillin-sulfuric acid method. The extracts were mixed with vanillin (8%, w/v) and sulfuric acid (72%, w/v) and then incubated at 60°C for 10 min. Being cooled in ice water for 15 min, the absorbance was measured at 538 nm. Quillaja saponin was used as a reference standard and the content of total saponins was expressed as Quillaja saponin equivalents (*μ*g/mg extract) [20]. The total RT saponins extracts were 23.998 mg/g.

### 2.3. Preparation of Oxyhemoglobin (OxyHb)

OxyHb was prepared based on the previous report with slight modification [21]. Briefly, arterial blood collected from Kunming mouse with heparin was centrifuged at 2,500 g for 15 min. After being washed 3 times with saline solution, erythrocytes were lysed with methylbenzene and centrifuged at 15,000 g for 20 min. OxyHb solution was adjusted to 3 *μ*mol/L and stored at −80°C.

### 2.4. Animals

The animal use and care protocols were approved by Institutional Animal Care and Use Committee (IACUC) of Xinxiang Medical University. Forty adult male Kunming mice weighing from 18 to 20 g were purchased from Xinxiang Medical University. All animals were required to undergo institutional quarantine for 7 days prior to use. The environment for animal housing was equipped with controlled temperature (22 ± 3°C), humidity (40–70%), and a 12 h light/dark alternation. The mice were divided into three groups: sham group (*n* = 10), SAH group (*n* = 10), and preadministration RT saponins SAH group (*n* = 20), which was subdivided into 2 mg/kg/d and 3 mg/kg/d dose (based on Chinese pharmacopoeia) subgroup (*n* = 10), respectively. SAH group was injected with OxyHb. Pretreatment group was administered intragastrically with water containing RT saponins for 14 days before OxyHb injection.

### 2.5. Mouse SAH Model

SAH was performed using the model reported by Shi et al. [21]. Briefly, prone positioned anesthetized animals were dissected through the parietal sagittal incision to expose cranial bones. After penetrating transparent atlanto-occipital membrane with a 30 ga needle, a 23 ga needle without point was inserted into skull in the depth of 1.5 mm at the junction position of the sagittal suture 2 mm and of sutura coronaria 1 mm. 50 *μ*L (150 *μ*mol/L) of OxyHb was injected into subarachnoid space at pressure equal to the mean arterial blood pressure. The needle was removed after 10 min of an infusion. Sham-operated rats were injected with physiological salt solution instead. At 24 h after SAH, all the mice blood was collected from heart. Five mice were perfused through the left cardiac ventricle with 0.9% saline solution to dissect cortex sections and then stored at −80°C. The others (*n* = 5) were perfused with 4% paraformaldehyde in phosphate-buffered saline (PBS). Removed brain tissue was fixed in 4% paraformaldehyde for 48 h and then embedded in paraffin.

### 2.6. Neurological Functions Assessment

The animal neurological behavior and function were evaluated by the Garcia scoring system with slight modification, including (1) spontaneous activity, (2) symmetry of limb movement, (3) climbing, (4) body proprioception, (5) movement of forelimbs, and (6) response to vibrissae touch (score scale: 0–3 each) [22].

### 2.7. Lactate Dehydrogenase (LDH) Assay from Brain Cortex

The supernatant of all the samples was collected after homogenate and the LDH content was determined using an LDH assay kit according to the manufacturer's instructions (Nanjing Institute of Jiancheng Biological Engineering, China).

### 2.8. SOD, MDA, NO, and iNOS Assay

The SOD, MDA, NO, and iNOS activities of cortex and serum were detected using SOD, MDA, NO, and iNOS assay kit, respectively, according to the manufacturer's instructions (Nanjing Institute of Jiancheng Biological Engineering, China).

### 2.9. Immunohistochemistry for p53 and p-p38 Antigen

0.3% hydrogen peroxide was used to block endogenous peroxidase activity for 15 min at room temperature. The sections were incubated in 0.01 M, pH 6.5 sodium citrate buffer for 20 min at 95°C. After being blocked with 10% normal goat serum for 1 h at room temperature, the slides were subsequently incubated overnight with anti-p53 (at a dilution of 1 : 100, sc-6243, Santa Cruz) and anti-p-p38 antibody (at a dilution of 1 : 100, sc-101759, Santa Cruz), then incubated with SPlink Detection Kits (SP-9001, Zymed, USA), and counterstained with DAB (ZLI-9032, Zhongshan Golden Bridge Biotechnology Co., Ltd., China). Quantitative evaluation was measured using IDA-2000 software (Beijing Konghai Technology Company, China). At least 10 visual fields were captured and more than 500 cells were counted.

### 2.10. Statistical Analysis

The statistical analysis was performed using the Statistical Package for the Social Sciences (SPSS Inc., Chicago, IL) program. All data were reported as means ± SD of three independent experiments. The physiological variables were analyzed by one-way ANOVA followed by LSD multiple comparison post hoc analysis. The neurological scores were compared by Kruskal-Wallis nonparametric test followed by multiple comparison procedures by Duncan's method. For all comparisons, *P* < 0.05 was considered statistically significant.

## 3. Results

### 3.1. RT Saponins Protection Neuronal Cells in the Cortex after SAH

**Figure 1:**
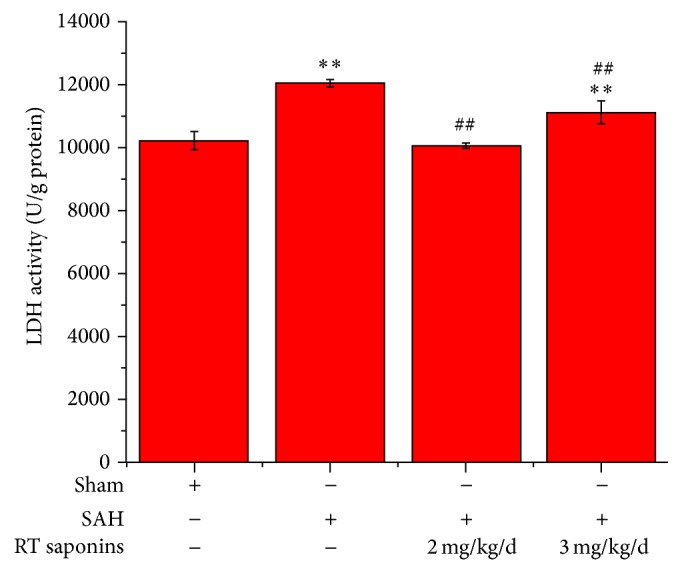
RT saponins decreased LDH activity after SAH. LDH activity was detected at 24 h after SAH with RT saponins pretreatment (2 mg/kg/d and 3 mg/kg/d). There is significantly decreased LDH level at 2 mg/kg/d RT saponins treatment. Data are expressed as the mean ± SD of three independent experiments. ^∗∗^ *P* < 0.01 versus sham; ^ΔΔ^ *P* < 0.01 versus SAH.

**Figure 2:**
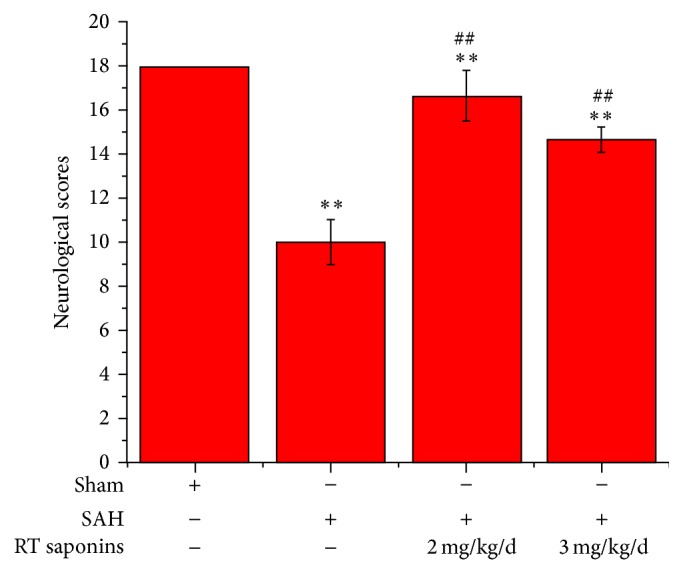
RT saponins increased neurological scores after SAH. Evaluation of neurological deficits by using Garcia scoring system at 24 h of OxyHb-induced SAH mice after RT saponins pretreatment at 2 mg/kg/d and 3 mg/kg/d dosage. There are significant increasing neurological scores after RT saponins pretreatment, especially at 2 mg/kg/d dosage. Values are expressed as mean ± SD of triplicate samples. ^∗∗^ *P* < 0.01 versus sham; ^ΔΔ^ *P* < 0.01 versus SAH.

LDH activity is the most widely used marker in cytotoxic studies. Using this assay, we detected a neuroprotective role of RT saponins after SAH (Figure 1). Contrary to peaked LDH levels in SAH group (*P* < 0.01 versus sham), RT saponins significantly decreased LDH activity (*P* < 0.01 versus SAH). Of interest, LDH activity returned to control level after RT saponins pretreatment with 2 mg/kg/d dosage, but 3 mg/kg/d RT saponins did not. Consistent with the LDH activities, the neurological score in SAH group was significantly lower than that of sham group (*P* < 0.01 versus sham), whereas the improved neurological scores were observed in animals pretreated with crude saponins group, especially 2 mg/kg/d group, indicating that RT saponins rescued neuronal injury (*P* < 0.01 versus sham, *P* < 0.01 versus SAH) (Figure 2).

### 3.2. The Effect of RT Saponins on SOD Activity after SAH

**Figure 3:**
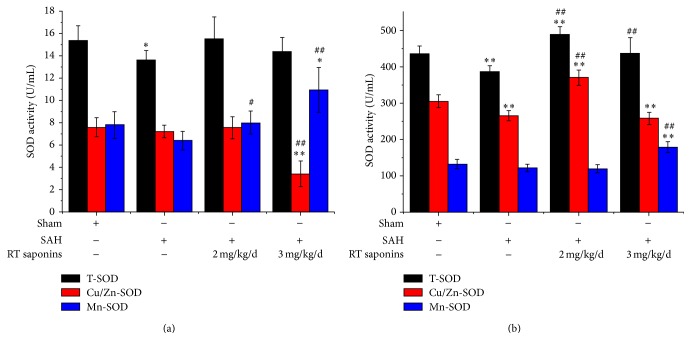
RT saponins increased SOD activity after SAH at 24 h. (a) After pretreatment with RT saponins (2 mg/kg/d and 3 mg/kg/d), cortexes were collected after 24 h of SAH. Significantly decreased Cu/Zn-SOD level was observed at 3 mg/kg/d RT saponins group, whereas dramatically increased Mn-SOD activities were detected after RT saponins treatment with 2 mg/kg/d and 3 mg/kg/d dosage; (b) after SAH, serum was used to detect T-SOD, Cu/Zn-SOD, and Mn-SOD activities. Contrary to decreased T-SOD and Cu/Zn-SOD levels after SAH, T-SOD and Cu/Zn-SOD levels were significantly increased only after 2 mg/kg/d RT pretreatment, whereas 3 mg/kg/d RT saponins significantly increased Mn-SOD acitivity. All the experiments were performed as described in Materials and Methods. Data are expressed as the mean ± SD of three independent experiments. ^∗∗^ *P* < 0.01 versus sham; ^Δ^ *P* < 0.05 versus SAH; ^ΔΔ^ *P* < 0.01 versus SAH.

In brain cortex and serum, significantly decreased total SOD (T-SOD) activity was observed in SAH group (*P* < 0.01 versus sham), which can be rescued by RT saponins to normal level (*P* > 0.05 versus sham), especially in serum showing about 1.12-fold sham and 1.26-fold SAH (*P* < 0.01 versus sham, *P* < 0.01 versus SAH) (Figure 3). As for Cu/Zn-SOD activity, the level was stable in sham, SAH, and 2 mg/kg/d RT saponins groups, whereas RT saponins at 3 mg/kg/d significantly inhibited Cu/Zn-SOD activity in cortex (about 0.45-fold sham and 0.47- fold SAH) (*P* < 0.01 versus sham, *P* < 0.01 versus SAH) (Figure 3(a)). On the contrary, in serum, Cu/Zn-SOD activity was significantly decreased after SAH (*P* < 0.01 versus sham), whereas only RT saponins at 2 mg/kg/d pretreatment can significantly increase Cu/Zn-SOD activity, which was about 1.2-fold sham and 1.4-fold SAH (*P* < 0.01 versus sham, *P* < 0.01 versus SAH) (Figure 3(b)). The trend of Mn-SOD activities both in cortex and in serum was similar (Figure 3), showing the dose dependent manner in RT saponins pretreatment groups, particularly in 3 mg/kg/d group (*P* < 0.01 versus sham, *P* < 0.01 versus SAH).

### 3.3. The Effect of RT Saponins on MDA after SAH

**Figure 4:**
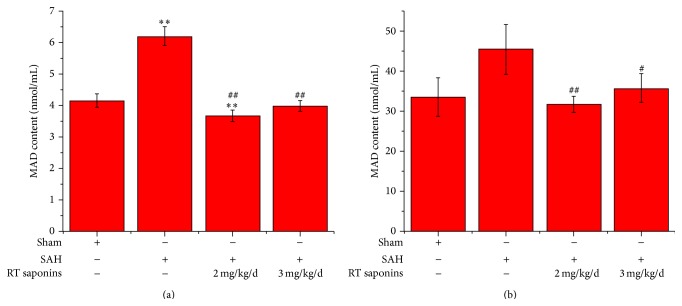
RT saponins inhibition lipid peroxidation after SAH. MDA assay was used to assess lipid peroxidation. (a) Effect of RT saponins at 2 mg/kg/d and 3 mg/kg/d on MDA content after SAH. Brain cortex was collected at 24 h after SAH. There is a significant difference between SAH and RT saponins treatment group; (b) MDA content in serum after SAH. A significant difference was shown in RT saponins treatment group compared with SAH. Data are expressed as the mean ± SD of three independent experiments. ^∗∗^ *P* < 0.01 versus sham; ^Δ^ *P* < 0.05 versus SAH; ^ΔΔ^ *P* < 0.01 versus SAH.

Figure 4 illustrated that MDA level both in cortex and in serum reached peak level after SAH (*P* < 0.01 versus sham). Once pretreated with RT saponins, MDA was significantly decreased to normal level both in brain tissue and in serum, indicating that RT saponins either at 2 mg/kg/d or at 3 mg/kg/d can significantly reduce free radical-mediated injury (*P* < 0.05 versus SAH).

### 3.4. The Effect of RT Saponins on iNOS Activity after SAH

**Figure 5:**
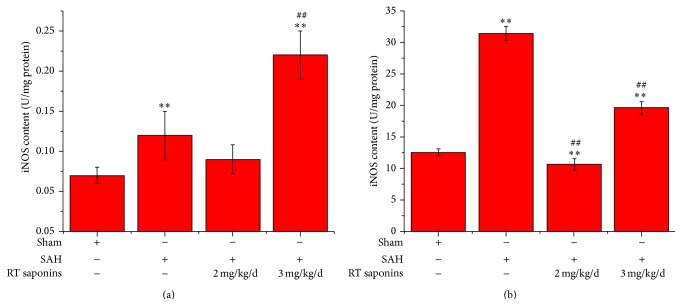
RT saponins at 2 mg/kg/d significant inhibition of iNOS activity. (a) Decreased iNOS activity was observed in RT saponins at 2 mg/kg/d in brain cortex at 24 h of SAH; (b) iNOS activities in serum were lower than that of sham when treated with 2 mg/kg/d RT saponins after SAH. Data are expressed as the mean ± SD of three independent experiments. ^∗∗^ *P* < 0.01 versus sham; ^ΔΔ^ *P* < 0.01 versus SAH.

After SAH, iNOS content was determined using a validated NOS detection assay (Figure 5). Both in brain tissue and in serum, significantly increased iNOS activities after SAH (*P* < 0.01 versus sham) can be rescued by pretreatment with 2 mg/kg/d RT saponins (*P* < 0.01 versus sham, *P* < 0.01 versus SAH). However, iNOS level in 3 mg/kg/d RT saponins group was higher than that of SAH group (*P* < 0.01 versus sham, *P* < 0.01 versus SAH).

### 3.5. The Effect of RT Saponins on NO after SAH

**Figure 6:**
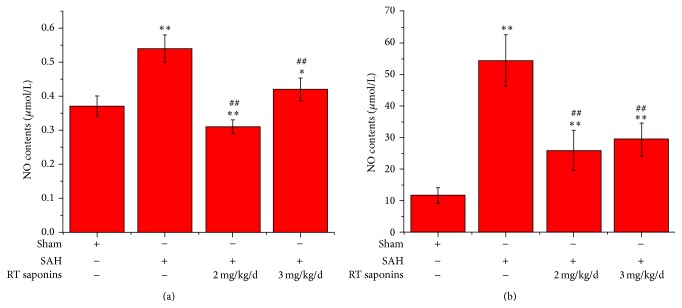
RT saponins at 2 mg/kg/d reduced NO content after SAH. (a) NO content was lower in RT saponins at 2 mg/kg/d than that of sham cortex at 24 h after SAH, and significant difference was observed; (b) NO content in serum was lessened after RT saponins treatment but not to normal level. Data are expressed as the mean ± SD of three independent experiments. ^∗^ *P* < 0.05 versus sham; ^∗∗^ *P* < 0.01 versus sham; ^ΔΔ^ *P* < 0.01 versus SAH.

As shown in Figure 6, after SAH, NO content reached peak level both in cortex and in serum (*P* < 0.01 versus sham). Pretreatment of RT saponins at 2 mg/kg/d only significantly reduces brain NO content to basal level (*P* < 0.01 versus sham, *P* < 0.01 versus SAH) (Figure 6(a)), whereas 3 mg/kg/d RT saponins had a medium effect on NO level both in cortex and in serum, and significant difference was observed (*P* < 0.01 versus sham, *P* < 0.01 versus SAH) (Figure 6).

### 3.6. RT Saponins Inhibition of p-p38 Expression after SAH

**Figure 7:**
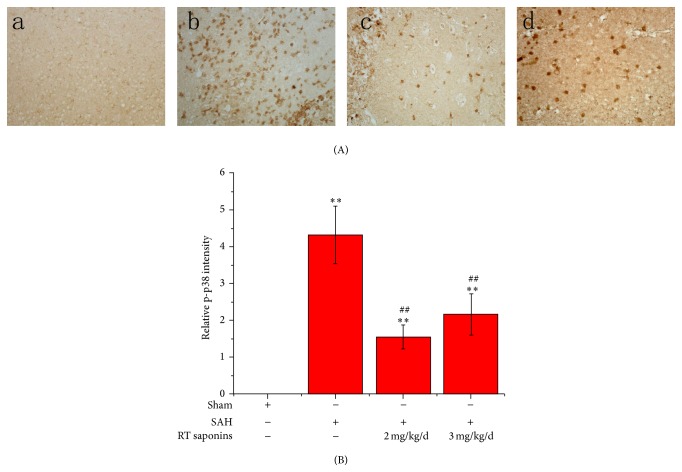
RT saponins inhibition of p-p38 expression. (A) Assessment of p-p38 activation following treatment with 2 mg/kg/d and 3 mg/kg/d RT saponins by using immunohistochemical assay (×400); (B) quantification of p-p38 level by densitometry. At least 10 visual fields were captured and more than 500 cells were counted. The quantification represents means and standard deviations of results from three independent experiments. ^∗∗^ *P* < 0.01 versus sham; ^ΔΔ^ *P* < 0.01 versus SAH.

To investigate the alteration of p-p38 after SAH, p-p38 protein was measured by immunohistology. As shown in Figure 7(A), in normal brain cortex, no p-p38 immunoreactivity was observed, whereas robust p-p38 expression was found after SAH. Following pretreatment with RT saponins, moderate p-p38 expression was observed especially in 2 mg/kg/d dose group. When quantified, the figures were 4.33 ± 0.78 in SAH, compared to 1.54 ± 0.32 and 2.16 ± 0.56 in RT saponins pretreatment groups. The decrease of p-p38 in RT saponins pretreatment groups was approximately 0.36- to 0.50-fold SAH. There were significant differences in quantity of p-p38 protein between SAH and RT saponins treatment groups (*P* < 0.01 versus SAH) (Figure 7(B)).

### 3.7. RT Saponins Inhibition of p53 Expression after SAH

**Figure 8:**
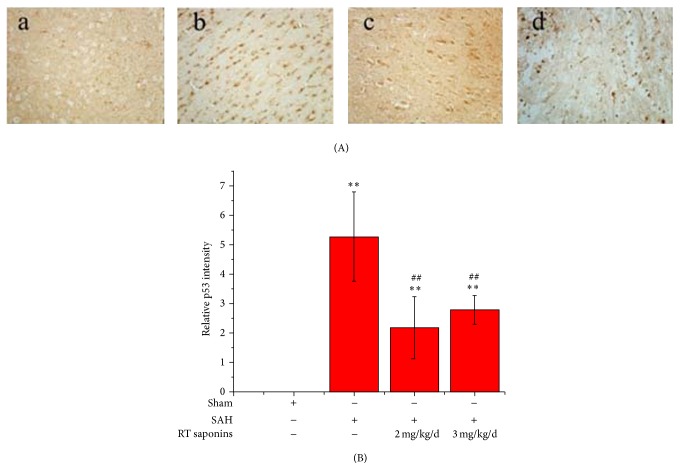
RT saponins inhibition of p53 expression after SAH. (A) Evaluation of p53 expression in SAH mice by immunohistochemical assay (×400). After RT saponins pretreatment, positive p53 staining in SAH was decreased; (B) quantification of p53 level by densitometry. At least 10 visual fields were captured and more than 500 cells were counted. The quantification represents means and standard deviations of results from three independent experiments. ^∗∗^ *P* < 0.01 versus sham; ^ΔΔ^ *P* < 0.01 versus SAH.

Similar to the changes seen with p-p38 protein, no p53 positive signal was observed in normal brain, whereas robust p53 expression was found after SAH (Figure 8). Following pretreatment with RT saponins, moderate p53 expression was observed especially in 2 mg/kg/d dose group. When quantified, the figures were 5.2763 ± 1.51 in SAH, compared to 2.1859 ± 1.058 and 2.7904 ± 0.493 in RT saponins pretreatment groups. The decrease of p53 in RT saponins pretreatment groups was approximately 0.41- to 0.53-fold SAH. There were significant differences in quantity of p53 protein between SAH and RT saponins treatment groups (*P* < 0.01 versus SAH) (Figure 8(B)).

## 4. Discussion

Recently, SAH progression has been roughly divided into two phases: early brain injury (EBI) (the first 72 h of the SAH) and delayed cerebral ischemia (DCI). More importantly, recent advances in SAH research have challenged the prevailing notion, which overlooked the role of EBI in the improvement of outcomes in patients after SAH [23]. We previously reported that interference EBI progression might be an optimal approach in SAH [16]. Therefore, RT saponins were used to investigate the mechanisms in the treatment of SAH at EBI. In the present study, we found that RT saponins at 2 mg/kg/d have stronger neuroprotective effects than that at 3 mg/kg/d through decreasing iNOS and NO level, increasing SOD activities, and inhibition of p-p38 and p53 expression.

SOD is effective against vasospasm by decreasing oxidative injury after SAH; however, neither intracisternal nor intrathecal administration of exogenous SOD can ameliorate vasospasm, indicating that how to stimulate endogenous SOD activity is crucial for outcomes of SAH [24–26]. In the present study, a significant downregulated SOD activity both in brain tissue and in serum after SAH can be rescued to normal level by pretreatment with RT saponins, indicating that stimulated endogenous SOD activity by RT saponins was a systemic effect. As the isoforms of SOD, the appropriate levels of Mn-SOD and Cu/Zn-SOD might predict neuroprotective effect, due to mutation of Mn-SOD showing a short life span with elevated ROS, and a strong downregulation of Cu/Zn-SOD preceding neuronal degeneration [7, 27–29]. In line with these findings, contrary to the lowest Cu/Zn-SOD levels in RT saponins (3 mg/kg/d) pretreatment group, our data showed* in vivo* evidence that decreased T-SOD and Mn-SOD activity can be restored to basal level both in brain and in serum after pretreatment with 2 mg/kg/d RT saponins, indicating that RT saponins mainly stimulated Mn-SOD activity. Based on these reports, we speculated that Mn-SOD may be the first line to scavenge ROS generated in mitochondria after RT saponins treated SAH either in the serum or in the brain tissue or in both. As a result, lipid peroxidation, which alters membrane fluidity and permeability, can be arrested by RT saponins [30].

During EBI after SAH, NO levels have been roughly divided into three phases: phase I (decrease in NO level till 60 min after SAH), phase II (recovery to baseline from 1 to 6 h after SAH), and phase III (increase in NO level from 24 h till 72 h) [31]. It is particularly noteworthy that contrary to the protective role of NO at phases I and II, an increase in NO level produced by iNOS at phase III is destructive for the compromised brain, which has close relation with oxidative damage to cell membranes, axonal degeneration, pathogenesis of delayed vasospasm, and the poor outcomes [32, 33]. In line with previous reports that significantly elevated iNOS and NO metabolites were observed in EBI following SAH [34, 35], RT saponins at 2 mg/kg/d can, however, significantly lessen the NO and iNOS levels, demonstrating that RT saponins might protect neuronal cell against injury by arresting cerebral vasospasm development through inhibition of ROS and RNS levels [36, 37].

Several groups demonstrated oxidative stress induced apoptosis was mediated through the activation of p38 pathway. Currently, many free radical scavenging compounds, antiapoptotic therapies focusing on p38 and p53, have been tested in experimental and clinical trials for the treatment of SAH [38–44]. We further detected p-p38 and p53 expression after RT saponins pretreatment. RT saponins, especially at 2 mg/kg/d, can significantly inhibit p-p38 expression, which has been found to regulate not only a variety of proapoptotic proteins, including p53, Bim, and BAX, but also antiapoptotic proteins, such as Bcl-2 and Bcl-xl after SAH. p53 mediated apoptosis was a universal phenomenon after SAH. Once p53 is activated, it simultaneously promotes prooxidant enzymes and suppresses antioxidant genes to induce oxidative stress, including* MnSOD* [7, 12]. However, these insults occurred at 24 hours after SAH can be attenuated by RT saponins, demonstrating that RT saponins inhibited p38/p53 mediated cell death pathway to protect neural cells against oxidative insults.

Taken together, our results demonstrated that the neuroprotection of RT saponins is a multiple target process. Meanwhile, as this study is based on mixed RT saponins components, great effects should be made to better identify which saponins play the roles in SAH. Hopefully, these researches will reveal new therapeutic avenues that can be exploited in combination with anti-EBI medications.

## 5. Conclusions

In summary, one of the attractive strategies in current SAH therapy is to block EBI progression; however, there is no effective clinical therapy from either preventive or therapeutic angles, partly due to the complex signaling network after SAH [45]. Therefore, pharmacological agents from traditional medical herbs might be optional approaches due to their multiple targets and less side effect properties. In line with RT saponins antioxidant activities, RT saponins at 2 mg/kg/d can significantly stimulate systemic endogenous SOD level and decreasing iNOS activity. Meanwhile, downregulated p-p38 and p53 have been observed after RT saponins pretreatment. These findings indicated that RT saponins enhance neuroprotective effects by deducing iNOS activity, normalizing SOD level, and inhibiting p-p38 and p53 expression, hence offering significant therapeutic implications for SAH.
